# Association between obesity and bone mineral density in middle-aged adults

**DOI:** 10.1186/s13018-022-03161-x

**Published:** 2022-05-14

**Authors:** Yue Li

**Affiliations:** Department of Orthopaedics, Tongde Hospital of Zhejiang Province, Hangzhou, 310012 Zhejiang China

**Keywords:** Bone mineral density, Obesity, Body mass index, NHANES

## Abstract

**Background:**

The relationship between obesity and bone mineral density (BMD) varies in different studies. Our aim in this study was to explore the association between obesity (body mass index ≥ 30) and BMD among adults 40–59 years of age.

**Methods:**

This study was conducted on a sample of 2218 participants (986 men and 1232 women) aged 40 to 59 years from the National Health and Nutrition Examination Survey 2011–2018. The independent variable was body mass index (BMI). The outcome variable was lumbar BMD. The associations of BMI with lumbar BMD were examined using multivariable linear regression models.

**Results:**

BMI was positively associated with lumbar BMD after adjusting for other covariates [*β* 0.006; 95% confidence interval (CI) 0.003–0.008]. An inverted U-shaped association between BMI and lumbar BMD was further identified, with the point of infection at approximately 50 kg/m^2^. In the subgroup analyses, the relationship between BMI and lumbar BMD in women and blacks was an inverted U-shape.

**Conclusion:**

Based on the results, it may be beneficial to appropriately increase BMI to promote BMD. However, considering the inverted U-shaped association, excessive BMI may be harmful to bone health in women and blacks.

## Introduction

Osteoporosis is a systemic skeletal metabolic disorder characterized by low bone mass density (BMD) and microarchitectural deterioration of bone tissue, with an increased risk of fragility fractures [1]. Osteoporosis and fragility fractures increase the burden not only on individual subjects but also on health care systems [2].

Obesity has become a serious public health problem worldwide, and obesity is associated with several medical conditions [3]. Body mass index (BMI) is used as a metric for assessing obesity, which exhibits good resolution among individuals of different ages, sexes and races [4]. High BMI is closely associated with BMD in both men and women. However, studies on the relationship between high BMI and BMD have found conflicting results. Some studies have demonstrated that high BMI is protective for osteoporosis [5], while an increasing amount of data seems to contradict this finding [6]. Our aim in the study was to evaluate the association between obesity and BMD among adults 40–59 years of age using a population-based database.

## Methods

### Study population

The National Health and Nutrition Examination Survey (NHANES) is a survey designed to provide large information about the nutrition and health conditions of the general population in America [7]. The data from 2011 to 2018 were combined in this paper. Of the 5731 individuals aged 40 to 59 years with available BMI and lumbar BMD data, 2218 were included in the final analysis after the exclusion of 332 subjects with cancer and 3181 non-obese subjects (BMI < 30). The survey protocols were approved by the ethics review board of the National Center for Health Statistics, and participants in the NHANES provided written consent.

### Variables

The key variables of the study were BMI (independent variable) and lumbar BMD (dependent variable). BMI was calculated as weight in kilograms divided by height in meters squared. Lumbar BMD was measured by dual-energy X-ray absorptiometry. Additional categorical variables were were included in the analysis: sex, race/ethnicity, level of education, vigorous recreational activities, and smoking behavior. Continuous covariates were also analyzed: age, poverty to income ratio, waist circumference, blood urea nitrogen, lumbar BMD, total protein, serum glucose, cholesterol, phosphorus, and total calcium. Detailed information on BMI, lumbar BMD, and covariates is publicly available from NHANES.

### Statistical analysis

All estimates were calculated accounting for NHANES sample weights. Weighted multivariate linear regression models and smooth curve fittings were used to evaluate the associations between BMI and lumbar BMD. Three models were implemented: Model 1, no adjustment for covariates; Model 2, adjusted for age and race/ethnicity; and Model 3, adjusted for all covariates. These models adhered to the Strengthening the Reporting of Observational Studies in Epidemiology (STROBE) statement [8]. All analyses were performed with EmpowerStats (http://www.empowerstats.com) and R package (http://www.Rproject.org).

## Result

A total of 2218 participants, 40–59 years of age, were included in the analysis, with the weighted characteristics of the participants subclassified based on BMI quartiles (Q1: 30.0–31.8 kg/m^2^; Q2: 31.9–34.2 kg/m^2^; Q3: 34.3–38.4 kg/m^2^; and Q4: 38.5–65.8 kg/m^2^), as shown in Table 1. There were significant differences in baseline characteristics among the BMI quartiles, except for some of the covariates: age, sex, the income to poverty ratio, smoking behavior, total protein, and phosphorus.

**Table 1:** Weighted characteristics of study population based on BMI quartiles

BMI (kg/m^2^)	Q1 (30.0–31.8)	Q2 (31.9–34.2)	Q3 (34.3–38.4)	Q4 (38.5–65.8)	*P* value
Age (years)	49.31 ± 5.60	49.79 ± 5.95	49.53 ± 5.75	49.37 ± 5.58	0.4894
Sex (%)					0.7802
Men	61.59	45.05	46.18	44.59	
Women	57.31	54.84	53.82	55.41	
Race/ethnicity (%)					< 0.0001
Non-Hispanic white	64.49	64.43	57.08	63.22	
Non-Hispanic black	11.64	11.87	19.43	19.89	
Mexican American	10.38	10.15	12.50	8.77	
Other race/ethnicity	13.49	13.55	10.99	8.11	
Waist circumference (cm)	104.68 ± 6.54	109.54 ± 7.15	115.13 ± 7.90	130.67 ± 12.06	< 0.0001
Level of education (%)					0.0044
Less than high school	15.96	15.07	15.35	12.06	
High school	19.60	27.61	26.23	21.64	
More than high school	64.43	57.33	58.42	66.30	
Income to poverty ratio	3.18 ± 1.62	3.17 ± 1.61	3.00 ± 1.62	3.06 ± 1.60	0.2449
Vigorous recreational activities (%)					< 0.0001
Yes	21.60	22.00	13.61	10.89	
No	78.40	78.00	86.39	89.11	
Smoked at least 100 cigarettes in life (%)					0.7802
Yes	42.69	45.05	46.18	44.59	
No	57.31	54.95	53.82	55.41	
Blood urea nitrogen (mmol/L)	5.01 ± 1.64	4.84 ± 1.61	4.63 ± 1.48	4.87 ± 2.11	0.0049
Total protein (g/L)	70.76 ± 3.95	70.88 ± 4.05	70.59 ± 4.57	70.51 ± 4.35	0.4786
Cholesterol (mmol/L)	5.36 ± 1.10	5.26 ± 1.04	5.16 ± 1.19	4.92 ± 0.97	< 0.0001
Phosphorus (mmol/L)	1.17 ± 0.17	1.18 ± 0.18	1.18 ± 0.18	1.18 ± 0.18	0.7604
Total calcium (mmol/L)	2.34 ± 0.08	2.33 ± 0.08	2.32 ± 0.09	2.31 ± 0.09	< 0.0001
Serum glucose (mmol/L)	5.93 ± 2.39	5.84 ± 2.36	6.33 ± 3.03	6.52 ± 2.71	< 0.0001
Lumbar BMD (g/cm^2^)	1.02 ± 0.16	1.02 ± 0.15	1.03 ± 0.16	1.08 ± 0.17	< 0.0001

Mean ± SD for continuous variables: the *P* value was calculated by the weighted linear regression model. % for categorical variables: the *P* value was calculated by the weighted chi-square test

The association between BMI and lumbar BMD was positive in all three regression models (Table 2): Model 1 (*β* 0.005; 95% confidence interval (CI) 0.004–0.006); Model 2 (*β* 0.004; 95% CI 0.003–0.006); and Model 3 (*β* 0.006; 95% CI 0.003–0.008). In subgroup analyses, with subgroups defined by sex, race/ethnicity and age, a positive correlation of BMI with lumbar BMD was found for both men (*β* 0.013; 95% CI 0.008–0.018; *P* < 0.001), women (*β* 0.003; 95% CI 0.000–0.006; *P* 0.044), whites (*β* 0.006; 95% CI 0.001–0.010; *P* 0.013), blacks (*β* 0.008; 95% CI 0.003–0.013; *P* 0.002), Mexican Americans (*β* 0.006; 95% CI 0.000–0.012; *P* 0.037) as well as in 40–49 years (*β* 0.008; 95% CI 0.004–0.011; *P* < 0.001) and 50–59 years (*β* 0.004; 95% CI 0.001–0.008; *P* 0.021), as reported in Table 2.

**Table 2:** Association between BMI (kg/m^2^) and lumbar bone mineral density (g/cm^2^)

	Model 1	Model 2	Model 3
	*β* (95% CI) *P* value	*β* (95% CI) *P* value	*β* (95% CI) *P* value
BMI (kg/m^2^)	0.005 (0.004, 0.006) < 0.001	0.004 (0.003, 0.006) < 0.001	0.006 (0.003, 0.008) < 0.001
BMI categories			
Q1 (30.0–31.8 kg/m^2^)	Reference	Reference	Reference
Q2 (31.9–34.2 kg/m^2^)	− 0.007 (− 0.026, 0.011) 0.437	− 0.009 (− 0.027, 0.010) 0.362	− 0.003 (− 0.023, 0.017) 0.774
Q3 (34.3–38.4 kg/m^2^)	0.003 (− 0.017, 0.022) 0.793	− 0.002 (− 0.021, 0.017) 0.829	− 0.007 (− 0.029, 0.016) 0.568
Q4 (38.5–65.8 kg/m^2^)	0.054 (0.035, 0.073) < 0.001	0.049 (0.030, 0.068) < 0.001	0.034 (0.002, 0.066) 0.038
Subgroup analysis stratified by sex			
Men	0.008 (0.005, 0.010) < 0.001	0.007 (0.005, 0.009) < 0.001	0.013 (0.008, 0.018) < 0.001
Women	0.003 (0.002, 0.004) < 0.001	0.003 (0.002, 0.004) < 0.001	0.003 (0.000, 0.006) 0.044
Subgroup analysis stratified by race/ethnicity			
Non-hispanic white	0.005 (0.003, 0.007) < 0.001	0.005 (0.003, 0.007) < 0.001	0.006 (0.001, 0.010) 0.013
Non-hispanic black	0.003 (0.001, 0.005) 0.004	0.004 (0.001, 0.006) 0.003	0.008 (0.003, 0.013) 0.002
Mexican American	0.005 (0.002, 0.008) < 0.001	0.005 (0.002, 0.008) < 0.001	0.006 (0.000, 0.012) 0.037
Other race/ethnicity	0.002 (− 0.001, 0.004) 0.171	0.003 (− 0.000, 0.005) 0.056	0.001 (− 0.004, 0.007) 0.598
Subgroup analysis stratified by age			
40–49 years	0.005 (0.004, 0.007) < 0.001	0.004 (0.002, 0.006) < 0.001	0.008 (0.004, 0.011) < 0.001
50–59 years	0.004 (0.002, 0.006) < 0.001	0.005 (0.003, 0.006) < 0.001	0.004 (0.001, 0.008) 0.021

Model 1: no covariates were adjusted. Model 2: age, sex, and race/ethnicity were adjusted. Model 3: age, sex, and race/ethnicity, level of education, poverty to income ratio, vigorous recreational activities, smoking behavior, waist circumference, blood urea nitrogen, total protein, serum glucose, cholesterol, phosphorus, and total calcium were adjusted

**Fig. 1:**
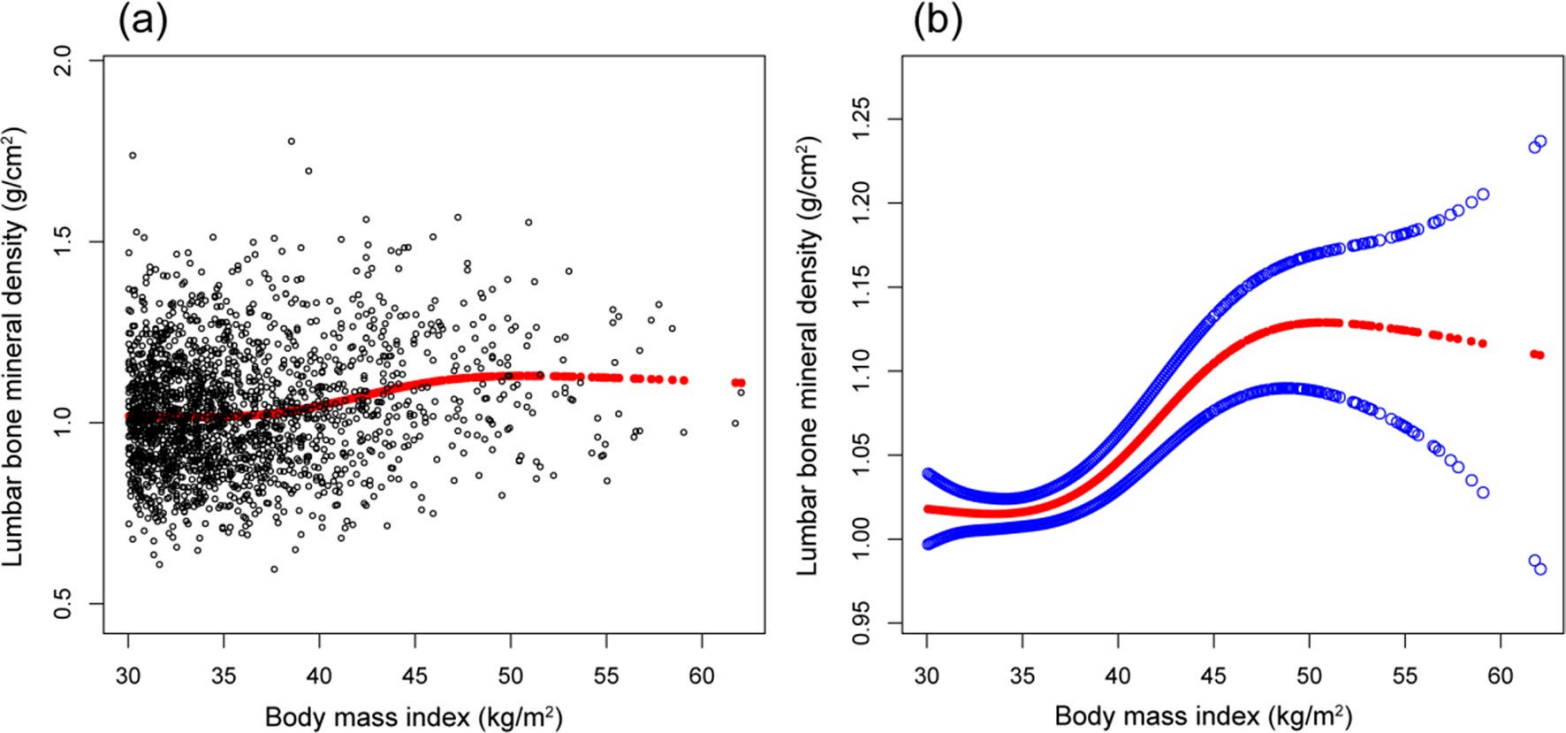
The associations between BMI and lumbar BMD. **a** Each black point represents a sample. **b** Solid red line represents the smooth curve fit between variables. Blue bands represent the 95% of confidence interval from the fit. Adjusted for age, sex, and race/ethnicity, level of education, poverty to income ratio, vigorous recreational activities, smoking behavior, waist circumference, blood urea nitrogen, total protein, serum glucose, cholesterol, phosphorus, and total calcium

**Fig. 2:**
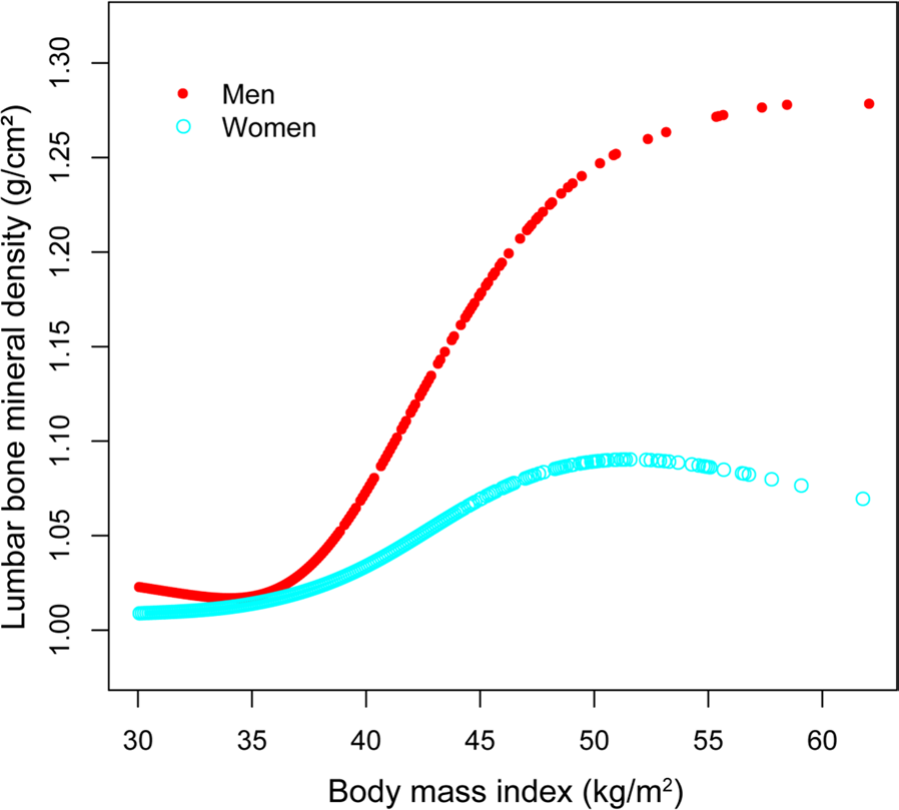
The association between BMI and lumbar BMD stratifified by sex. Age, and race/ethnicity, level of education, poverty to income ratio, vigorous recreational activities, smoking behavior, waist circumference, blood urea nitrogen, total protein, serum glucose, cholesterol, phosphorus, and total calcium were adjusted

**Fig. 3:**
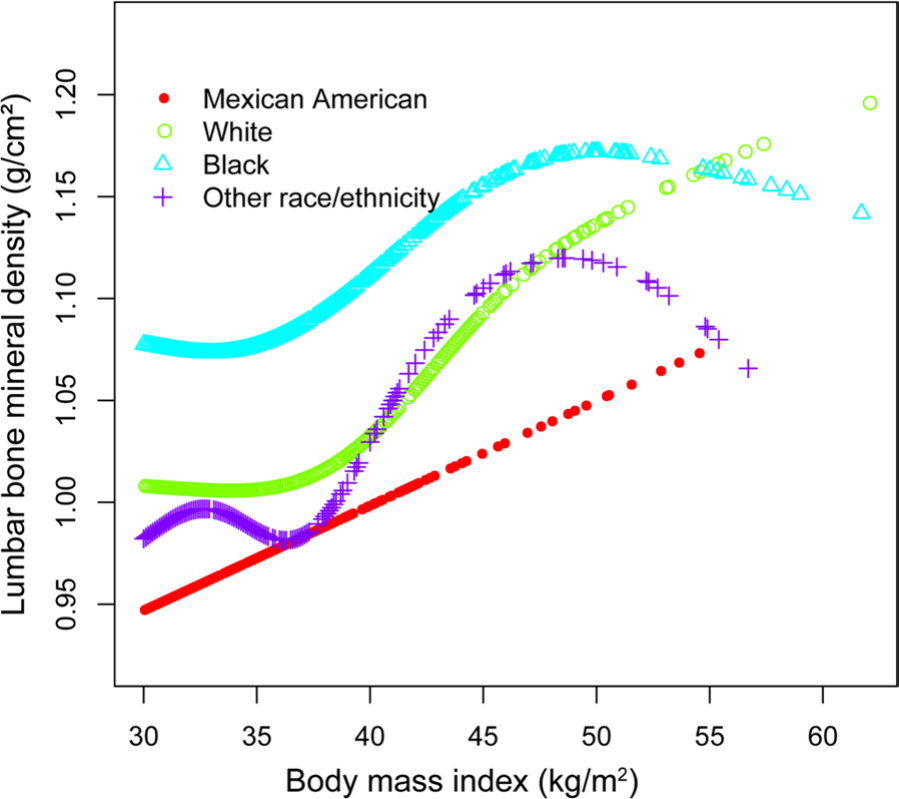
The association between BMI and lumbar BMD stratified by race/ethnicity. Age, sex, level of education, poverty to income ratio, vigorous recreational activities, smoking behavior, waist circumference, blood urea nitrogen, total protein, serum glucose, cholesterol, phosphorus, and total calcium were adjusted

**Fig. 4:**
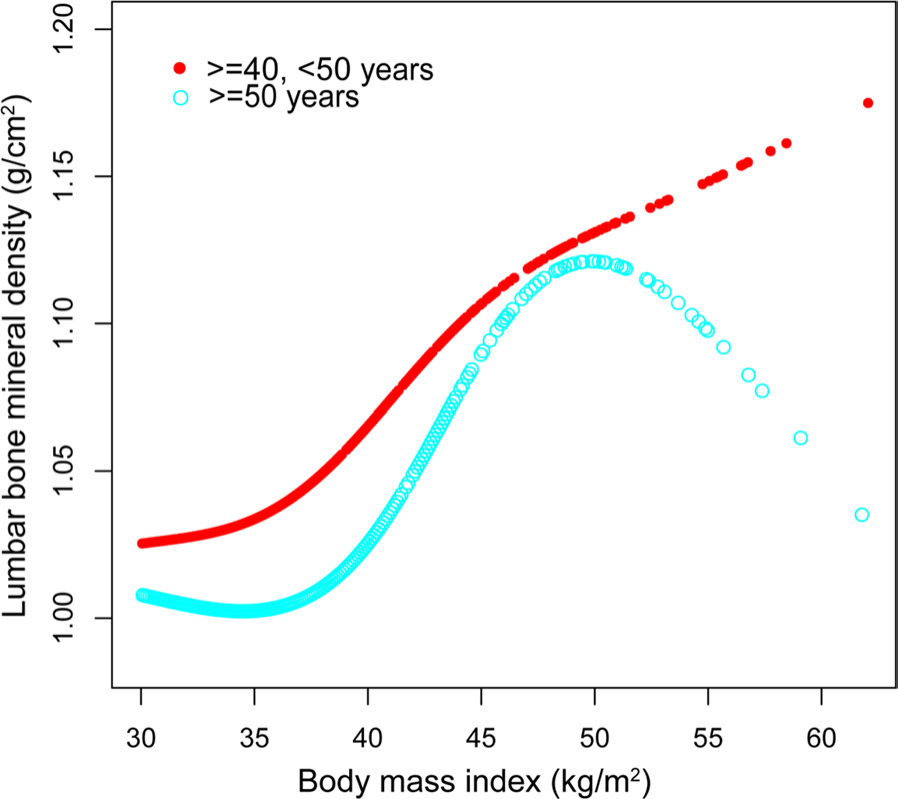
The association between BMI and lumbar BMD stratified by age. Sex, race/ethnicity, level of education, poverty to income ratio, vigorous recreational activities, smoking behavior, waist circumference, blood urea nitrogen, total protein, serum glucose, cholesterol, phosphorus, and total calcium were adjusted

Smooth curve fittings and generalized additive models used to characterize the nonlinear relationship between BMI and lumbar BMD are shown in Figs. 1, 2, 3 and 4. The graphical representation of the association between BMI and lumbar BMD was an inverted U-shaped and the point of infection was approximately 50 kg/m^2^. For a BMI < 50 kg/m^2^, an increase in BMI was associated with an increase in lumbar BMD; in contrast, for individuals with a BMI > 50 kg/m^2^, an increase in BMI was associated with a decrease in lumbar BMD. In subgroup analyses stratified by sex, race/ethnicity and age, the graphical relationship between BMI and lumbar BMD for women, blacks and elder (50–59 years) was an inverted U-shape.

## Discussion

This cross-sectional study involving 2218 obese adults aged 40–59 showed a significantly positive association between high BMI and lumbar BMD. Of note, we identified an inverted U-shaped association between high BMI and BMD.

Currently, clinical studies regarding the correlation between obesity and BMD are limited and controversial. A study from the US reported a significant positive association between obesity and BMD existed in elderly groups of both sexes [9]. In a study of 502 northern Chinese men, the total body and regional BMD in obese subjects were significantly higher than those of subjects with normal weight (*P* < 0.001) [10]. Similar results were reported by Silva et al. in postmenopausal women and reported a lower prevalence of osteoporosis at the lumbar spine and femoral neck in obese patients (*P* < 0.001) [11]. However, there is emerging literature that reports the opposite result. A study from India indicated that individuals with BMI ≥ 35 kg/m^2^ have a lower BMD than those with BMI ≥ 25–35 kg/m^2^. (0.723 gm/cm^2^ versus 0.762 gm/cm^2^; *P* 0.002) [12]. Another study from China based on 8365 adolescents aged 12–15 years concluded that obese subjects had a higher risk of having low BMD compared to subjects of normal weight for both sexes [13]. A retrospective study from Spain showed that obese females had an increased risk of proximal fractures compared with normal or underweight women (RR: 1.28; 95% CI 1.04–1.58; *P* 0.018) [14].

In our study, we identified an inverted U-shaped association between BMD and high BMI, with a point of infection at approximately 50 kg/m^2^. Excessive BMI may be harmful to bone health. In subgroup analyses, we found that the reason for this result was due to the findings for women, blacks and elder (50–59 years). There are sex differences in the relationship between BMI and lumbar BMD. The Busselton Healthy Aging Study showed that BMI is associated with reduced BMI in women but not in men [15]. One mechanism might explain why the lower BMD found in women and elder (50–59 years) BMI > 50 kg/m^2^ is the reduced estradiol. Menopause cause a quick increase in bone turnover, brings higher bone resorption and leads to bone loss [16, 17]. Racial differences in BMD have been described in adolescents and adults with normal weight and obesity in various studies [18–21], and differences in genetic risk factors, lifestyle and other factors may explain the race-specific differences. Further studies are required to clarify the association between BMI and BMD among individuals of the women and black individuals.

We used nationally representative data and performed subgroup analyses. Therefore, our results may be different from those previously reported. Moreover, it is important to acknowledge the limitations of the study. First, the study was a cross-sectional study, which limits the accuracy of the inference regarding the relationship between high BMI and lumbar BMD among middle-aged adults thus, further RCTs are necessary to strengthen evidence of the exact relationship between obesity and BMD. Second, there may be other confounding factors for which we did not adjust for such as the medical histories of the patients, such as diagnoses, drugs and procedures, which could affect the result [22, 23]. The application of additional available methodologies might help to identify potential confounders that we did not find in this study [24–26]. Third, participants with cancer or malignancy were not included in the study therefore, the conclusion of the research is not applicable to patients with those conditions.

## Conclusions

The findings revealed an inverted U-shaped association between BMI and lumbar BMD among obese middle-aged adults, suggesting that it may be beneficial to appropriately increase BMI levels to promote bone health. However, for women and blacks, excessively high BMI may may contribute to loss of BMD.

## Abbreviations

BMI: Body mass index
BMD: Bone mineral density
NHANES: National Health and Nutrition Examination Survey
